# Comparison of antimicrobial susceptibility testing methods for aztreonam-avibactam against NDM-producing Enterobacterales clinical isolates

**DOI:** 10.1128/jcm.00797-26

**Published:** 2026-10-12

**Authors:** Lina Rivas, Lorena Díaz, Valeria Quiroz, Jose R. W. Martinez, Camila Solar, Ayesha Khan, Katherine Soto, Pamela Rojas Soto, Maria-Paz Acuña, María L. Rioseco, Margareta Mühlhauser, Yehudit Bergman, Patricia García, José M. Munita, Pranita D. Tamma

**Affiliations:** 1 Genomics & Resistant Microbes Group (GeRM), Instituto de Ciencias e Innovación en Medicina (ICIM), Facultad de Medicina Clínica Alemana, Universidad del Desarrollo, Santiago, Chile; 2 Multidisciplinary Initiative for Collaborative Research on Bacterial Resistance (MICROB-R) 739773 , Santiago, Chile; 3 Surveillance, Epidemiology, and New Technologies for Infectious Emerging Threats (SENTINET), Santiago, Chile; 4 UC Irvine School of Medicine 12219, Irvine, California, USA; 5 Hospital Padre Hurtado, Santiago, Chile; 6 Pontificia Universidad Católica de Chile 28033, Santiago, Chile; 7 Hospital La Florida, Santiago, Chile; 8 Hospital Regional de Puerto Montt, Universidad San Sebastián 234475 , Puerto Montt, Chile; 9 Hospital Dipreca, Santiago, Chile; 10 The University of Pennsylvania School of Medicine 14640, Philadelphia, Pennsylvania, USA; Children's Hospital Los Angeles, Los Angeles, California, USA

**Keywords:** *Klebsiella pneumoniae*, *Escherichia coli*, *Enterobacter cloacae* complex, carbapenemases, antimicrobial resistance

## Abstract

Aztreonam-avibactam (ATM-AVI) is a promising therapeutic for New Delhi metallo-β-lactamase-producing Enterobacterales (NDM-E) infections. We evaluated the performance of disk diffusion and gradient diffusion methods compared with reference broth microdilution (BMD) for ATM-AVI susceptibility testing. A total of 245 non-duplicate NDM-E isolates were included, collected from unique patients at hospitals in Chile and the United States between 2022 and 2024. Nine ATM-AVI non-susceptible isolates from a separate collection were added to enrich for resistant isolates, yielding a total of 254 isolates. Reference BMD was performed according to Clinical and Laboratory Standards Institute (CLSI) criteria. ATM-AVI disk diffusion and two gradient diffusion methods (ETEST and MIC Test Strip) were evaluated against BMD using categorical agreement, essential agreement, and CLSI-defined error rates. All 245 NDM-E isolates were susceptible to ATM-AVI (MIC ≤4/4 µg/mL), with MIC₅₀/₉₀ values of 0.25/4 and 1/4 µg/mL, respectively. The collection consisted of *Klebsiella pneumoniae* (71%), *Enterobacter cloacae* complex (21%), and *Escherichia coli* (8%). Predominant NDM alleles included *bla*_NDM-1_ and *bla*_NDM-7_. Disk diffusion demonstrated 98% categorical agreement (250/254), with four minor errors. Both gradient diffusion methods achieved 99% categorical agreement (252/254), with no very major or major errors and two minor errors. Essential agreement was 67% for ETEST and 85% for MIC Test Strip; both methods tended to underestimate ATM-AVI MICs relative to BMD. ATM-AVI demonstrated robust *in vitro* activity against a contemporary NDM-E cohort. Disk and gradient diffusion methods are practical alternatives to BMD for clinical laboratories.

## INTRODUCTION

The prevalence of New Delhi metallo-β-lactamase-producing Enterobacterales (NDM-E) is increasing rapidly and, in many regions, exceeds that of *Klebsiella pneumoniae* carbapenemase producers (1). Infections caused by NDM-E are associated with high mortality, often exceeding 30% (1–3) largely due to limited therapeutic options (4). The β-lactam-β-lactamase inhibitor combination aztreonam-avibactam (ATM-AVI) represents an important therapeutic advance, with availability expanding across multiple regions worldwide (5).

Aztreonam is stable to hydrolysis by NDM enzymes but remains vulnerable to co-produced serine β-lactamases, such as extended-spectrum β-lactamases (6). Avibactam restores aztreonam activity by inhibiting serine β-lactamases. Regrettably, resistance to ATM-AVI has been reported among clinical NDM-E isolates (7, 8), underscoring the importance of confirmatory antimicrobial susceptibility testing (AST) prior to clinical use.

While reference broth microdilution (BMD) remains the standard for AST, it is labor intensive and not routinely feasible for many clinical laboratories. As newer agents, such as ATM-AVI, are generally unavailable on automated testing platforms, laboratories commonly rely on disk diffusion or gradient diffusion methods for susceptibility testing. However, the accuracy of these methods for NDM-E isolates is unclear. Accordingly, we evaluated the reliability of commercially available disk and gradient diffusion methods compared to reference BMD for ATM-AVI susceptibility testing using a contemporary collection of NDM-E clinical isolates.

## MATERIALS AND METHODS

### Cohort description

Clinical isolates were obtained from established biorepositories encompassing eight hospitals in Chile and The Johns Hopkins Hospital in the United States. At both central laboratories, all carbapenem-resistant gram-negative clinical isolates have been systematically archived since January 2019 and have undergone molecular characterization for carbapenemase genes.

For the present study, the primary cohort comprised 203 clinical NDM-E isolates collected between 2022 and 2024 from the eight participating hospitals in Chile (Fig. S1). To increase genetic diversity, minimize the potential influence of clonally related isolates, and broaden representation of NDM alleles, the cohort was supplemented with 42 NDM-E clinical isolates collected at The Johns Hopkins Hospital during the same period, yielding a total of 245 clinical isolates. Only one non-duplicate isolate per patient was included in the study. For patients from whom NDM-E were identified in multiple specimen types (e.g., both urine and blood), only the first recovered isolate was included. All isolates were stored at −80°C in glycerol until further testing.

Initial BMD testing demonstrated that all 245 isolates were susceptible to ATM-AVI (MIC ≤4 µg/mL) according to Clinical and Laboratory Standards Institute (CLSI) M100, 36th ed., criteria (9). To ensure adequate representation of ATM-AVI resistance for evaluation of susceptibility testing methods, nine additional non-susceptible isolates were included: five from the U.S. Centers for Disease Control and Prevention and U.S. Food and Drug Administration Antibiotic Resistance Isolate Bank and four from a clinical biorepository at the University of California, Irvine, bringing the total collection to 254 isolates. The study was approved by the institutional review boards of all participating institutions, with a waiver of informed consent.

### NDM confirmation

The presence of carbapenemase genes (*bla*_KPC_, *bla*_NDM_, and *bla*_VIM_) among the 254 isolates previously identified as containing *bla*_NDM_ genes through routine clinical testing was confirmed using conventional multiplex PCR, as previously described (10). The primers used for *bla*_NDM_ detection were NDM-F (5′-ATCCTCAACTGGATCAAGCAGG-3′) and NDM-R (5′-CGGTGATATTGTCACTGGTGTGG-3′). Amplification products were evaluated by agarose gel electrophoresis.

### Microbiological methods

Organism identification was performed by matrix-assisted laser desorption/ionization-time of flight (MALDI-TOF MS) using the VITEK MS system (bioMérieux), software version 1.1.3-230651337 and knowledge base version refkb-full-8.10.0-4. Frozen stocks were sub-cultured onto tryptic soy agar and incubated at 35°C overnight. Pure colonies were sub-cultured onto tryptic soy agar and incubated at 35°C overnight. Reference BMD, disk diffusion, and gradient diffusion were performed in parallel using the same standardized 0.5 McFarland inoculum. Agar-based methods (i.e., disk and gradient diffusion) were performed on Mueller-Hinton agar prepared from the same lot of medium powder and batch preparation.

All 254 isolates underwent duplicate testing by each susceptibility method evaluated (BMD, disk diffusion, ETEST, and MIC Test Strip). A third replicate was performed when duplicate results differed by more than one doubling dilution or, as applicable, yielded discordant categorical interpretations. For analysis, the highest MIC or, for categorical results, the more resistant interpretation was used.

Quality control (QC) testing was performed daily in accordance with CLSI and method-specific manufacturer recommendations. *Klebsiella pneumoniae* ATCC 700603 recommended by CLSI M100 and the gradient diffusion strip manufacturers was included in all experiments. Additional QC organisms included *Escherichia coli* ATCC 25922, as recommended by bioMérieux (Durham, NC, USA) and Liofilchem (Waltham, MA, USA), and *Pseudomonas aeruginosa* ATCC 27853, as recommended by Liofilchem. Modal ATM-AVI MICs were determined for each QC strain. ATM-AVI susceptibility was interpreted according to CLSI criteria: ≤4/4 µg/mL, susceptible; 8/4 µg/mL, intermediate; and ≥16/4 µg/mL, resistant (9). BMD reproducibility was defined as agreement within ±1 doubling dilution between replicate MICs.

### Reference broth microdilution

Reference BMD was performed in accordance with CLSI M07, 12th ed., criteria (11) using cation-adjusted Mueller-Hinton broth (BD, Sparks, MD, USA) and in-house-prepared panels containing ATM at concentrations ranging from ≤0.015 to ≥16 µg/mL in combination with a fixed AVI concentration of 4 µg/mL (Pfizer, New York, NY, USA). ATM was also tested alone at concentrations ranging from ≤0.06 to ≥64 µg/mL. Panels were stored at −80°C until use, incubated at 35°C in ambient air, and read after 16–20 h by two independent readers, with discrepancies adjudicated by a third reader.

### Disk diffusion

ATM-AVI disk diffusion testing (30/20 µg; Liofilchem, Waltham, MA, USA) was performed on Mueller-Hinton agar (BD Difco, Franklin Lakes, NJ, USA) in accordance with CLSI M02, 14th ed., criteria (12). Plates were incubated at 35°C in ambient air for 16–18 h. Zones of inhibition were measured independently by two trained readers, with discrepancies adjudicated by a third. Results were interpreted using CLSI M100 criteria (susceptible, ≥25 mm; intermediate, 22–24 mm; resistant, ≤21 mm) (9).

Categorical agreement and error rates for disk diffusion relative to BMD were evaluated according to CLSI M23, 6th ed., criteria (13). Categorical agreement was defined as concordance between methods in the assigned interpretive category (susceptible, intermediate, or resistant), with ≥90% considered acceptable. Very major errors were defined as isolates resistant by BMD but susceptible by disk diffusion (target ≤1.5%), major errors as susceptible by BMD but resistant by disk diffusion (target ≤3%), and minor errors as all other discrepancies (target ≤10%).

### Gradient diffusion

Two ATM-AVI gradient diffusion methods were evaluated: ETEST (bioMérieux, Durham, NC, USA) and MIC Test Strip (Liofilchem, Waltham, MA, USA) following manufacturers’ instructions. For both methods, ATM-AVI concentrations ranged from 0.016/4 to 256/4 µg/mL. Mueller-Hinton agar plates were inoculated using a sterile cotton swab dipped in a 0.5 McFarland inoculum and prepared with the same suspension used for BMD and disk diffusion. Plates were incubated at 35 ± 2°C in ambient air.

ATM-AVI MICs were read at the point of 100% growth inhibition at 16–20 h. MIC values were interpreted according to standard twofold (doubling) dilution increments, with values rounded up to the next highest concentration as needed. Essential agreement was defined as gradient diffusion MICs within ±1 doubling dilution of the modal BMD MIC (13). Categorical agreement and error rates relative to BMD were determined, as described above.

### Bias analysis

Bias analysis was performed to assess systematic directional differences in MIC results between the test and reference methods. In accordance with International Organization for Standardization guidance, positive and negative biases were defined as the proportions of isolates for which MICs determined by gradient diffusion were higher or lower, respectively, than those obtained by reference BMD relative to the total number of isolates within the corresponding evaluable range (14). Bias was calculated using the following formulas:


$$Positive\;bias\;(\%)=\frac{No.\;of\;isolates\;with\;higher\;MIC\;than\;BMD}{N}\times 100$$

$$Negative\;bias\;(\%)=\frac{\text{No. of isolates with lower MIC than BMD}}{N}\times 100$$

ΔBias (%)=Positive bias (%)−Negative bias (%)

## RESULTS

### Overall cohort

The baseline cohort comprised 245 consecutive NDM-E isolates prior to the inclusion of nine ATM-AVI non-susceptible isolates. Species distribution included *K. pneumoniae* (175/245, 71%), *Enterobacter cloacae* complex (51/245, 21%), and *Escherichia coli* (19/245, 8%). Isolates were recovered from blood (100/245, 41%), sterile tissue or fluid (86/245, 35%), urine (44/245, 18%), and respiratory specimens (15/245, 6%). PCR testing revealed that 9% (22/245) produced two carbapenemases: *bla*_NDM/KPC_ (19/245) and *bla*_NDM/VIM_ (3/245). All nine ATM-AVI non-susceptible isolates were *E. coli* and harbored only *bla*_NDM_ genes. Of note, whole-genome sequencing data generated through unrelated research studies were available for 92/245 (38%) baseline isolates and all six (100%) ATM-AVI-resistant isolates (Table S1), with methods and results described in the supplemental material.

### BMD results

**Fig 1:**
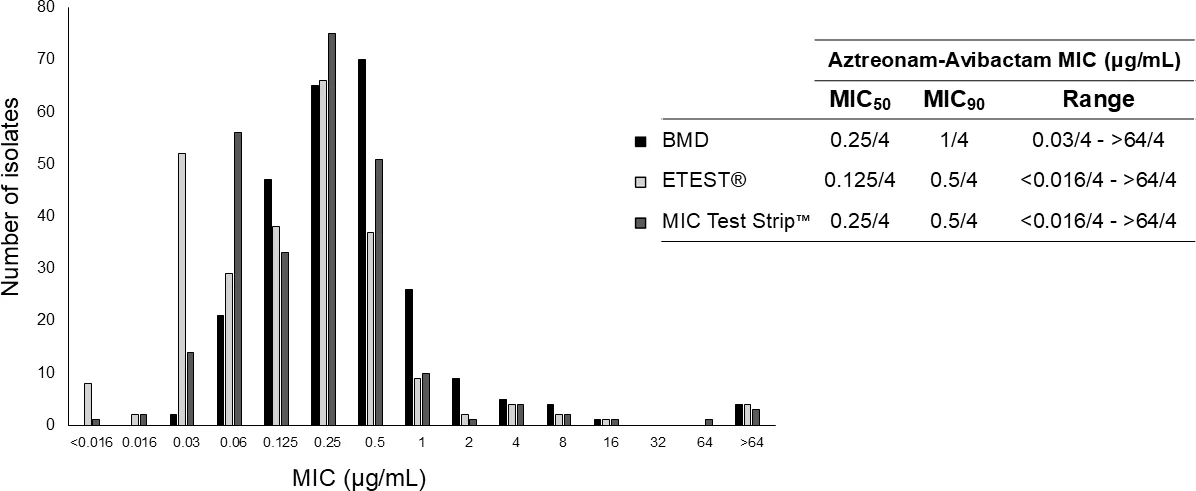
Comparison of aztreonam-avibactam (ATM-AVI) MIC values determined by reference broth microdilution.

Among the 245 baseline NDM-E isolates, all (245/245) were susceptible to ATM-AVI (Fig. 1). MICs ranged from 0.03/4 to 4/4 µg/mL, with MIC₅₀ and MIC₉₀ values of 0.25/4 and 1/4 µg/mL, respectively (Table 1). Of note, 86% (210/245) were resistant to ATM alone, with MIC₅₀ and MIC₉₀ values both >64 µg/mL. BMD reproducibility was 98% (240/245), with five isolates requiring a third replicate. Modal MICs for QC strains were 0.25/4 µg/mL for *K. pneumoniae*, 0.125/4 µg/mL for *E. coli*, and 4/4 µg/mL for *P. aeruginosa*. Identical MICs across replicates were observed in 57% (139/245) of isolates; variation between replicates did not affect categorical interpretation for any isolate. Modest interspecies differences were observed, with MIC₉₀ values of 1/4 µg/mL for *K. pneumoniae* and *E. cloacae* complex, compared with 0.25/4 µg/mL for *E. coli*. Among the nine ATM-AVI non-susceptible isolates, MIC values ranged from 8/4 to 64/4 µg/mL (Table S2). BMD reproducibility in this subset was 100% (9/9) (Fig. 1).

**TABLE 1:** MIC_50_ and MIC_90_ of aztreonam (ATM) and aztreonam-avibactam (ATM-AVI) among a cohort of 245 NDM-producing Enterobacterales isolates

Isolates	ATM (µg/mL)			ATM-AVI (µg/mL)		
	MIC_50_	MIC_90_	Range	MIC_50_	MIC_90_	Range
All isolates (*n* = 245)	>64	>64	0.016 to >64	0.25/4	1/4	0.03/4 to 4/4
*Klebsiella pneumoniae* (*n* = 175)	>64	>64	0.125 to >64	0.5/4	1/4	0.06/4 to 4/4
*Enterobacter cloacae* complex (*n* = 51)	64	>64	0.125 to >64	0.125/4	1/4	0.03/4 to 4/4
*Escherichia coli* (*n* = 19)	0.5	64	0.016 to >64	0.125/4	0.25/4	0.03/4 to 0.5/4

### Comparison of disk diffusion with BMD

**Fig 2:**
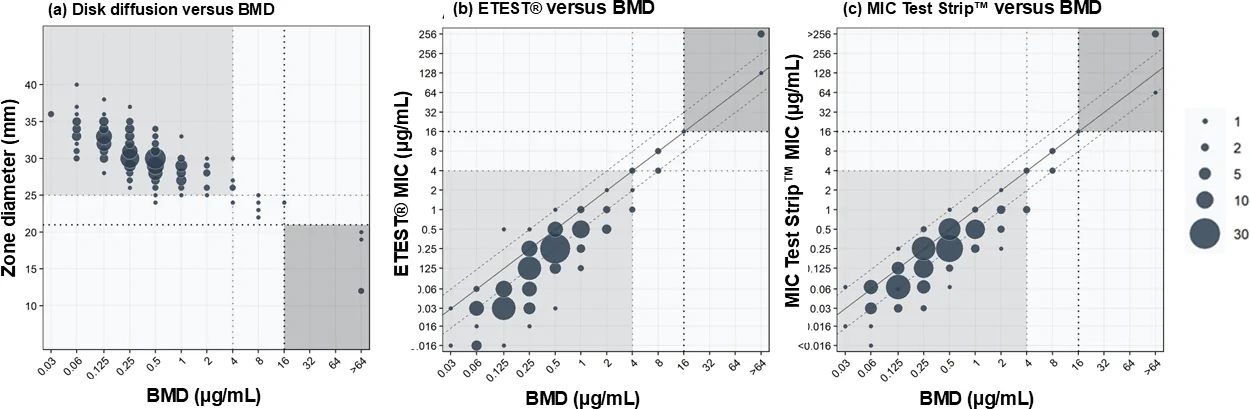
Comparison of aztreonam-avibactam (ATM-AVI) susceptibility between broth microdilution (BMD) and alternative testing methods. BMD ATM-AVI susceptibility results compared with (**a**) disk diffusion (30/20 µg), (**b**) ETEST, and (**c**) MIC Test Strip. Shaded regions represent susceptible (light gray: disk diffusion ≥25 mm, BMD/ETEST/ MIC Test Strip ≤4 µg/mL) and resistant (dark gray: disk diffusion ≤21 mm, BMD/ETEST/ MIC Test Strip ≥16 µg/mL) categorical zones for ATM-AVI according to CLSI M100, 36th ed. Solid diagonal denotes identity; dashed lines denote ±1 twofold dilution (essential agreement). Bubble size is proportional to the number of isolates.

The full cohort of 254 NDM-E, including the nine ATM-AVI-resistant isolates, was evaluated by disk diffusion and compared with reference BMD. Modal and median quality-control zone diameters were within established acceptable ranges. Overall categorical agreement between disk diffusion and BMD was 98% (250/254) (Table 2). Four minor errors occurred (2% [4/254]), with no major or very major errors observed (Fig. 2).

**TABLE 2:** Performance of disk diffusion, ETEST, and MIC Test Strips compared to reference broth microdilution results for aztreonam-avibactam against 254 NDM-producing Enterobacterales isolates

Antimicrobial susceptibility testing method	Categorical agreement	Very major errors	Major errors	Minor errors	Essential agreement
Disk diffusion	98% (250/254)	0% (0/9)	0% (0/254)	2% (4/254)	Not applicable
ETEST	99% (252/254)	0% (0/9)	0% (0/254)	1% (2/254)	67% (170/254)
MIC Test Strip	99% (252/254)	0% (0/9)	0% (0/254)	1% (2/254)	85% (217/254)

### Comparison of gradient diffusion with BMD

Comparison of gradient diffusion methods with BMD indicated high categorical agreement. Results from ETEST and MIC Test Strip showed 99% (252/254) agreement with BMD. No very major or major errors were observed; minor errors occurred for 1% (2/254) of isolates with both methods. Essential agreement with BMD was lower at 67% (170/254) for ETEST and 85% (217/254) for MIC Test Strip (Table 2). Notably, a greater proportion of data points fell below the line of agreement, indicating that both gradient diffusion methods tended to underestimate ATM-AVI MIC values relative to BMD (Fig. 2 and Fig. S2).

Bias analysis identified a systematic negative bias for both gradient diffusion methods relative to BMD, reflecting a tendency to yield lower ATM-AVI MICs than the reference method. The magnitude of negative bias was greater with ETEST (ΔBias, −31%) than with the MIC Test Strip (ΔBias, −23%), with the same directional bias observed across each of the three species (Table S3).

## DISCUSSION

Overall, ATM-AVI demonstrated excellent *in vitro* activity against a contemporary cohort of 245 clinical NDM-E isolates from unique patients in Chile and the United States. In addition, both disk diffusion and gradient diffusion methods showed high categorical agreement with reference BMD for ATM-AVI susceptibility testing, supporting their use as practical alternatives for clinical microbiology laboratories. Published evaluations of non-reference ATM-AVI susceptibility testing methods specifically against NDM-E remain limited; however, our findings are consistent with the only comparable study identified. In that evaluation of 134 NDM-E isolates, gradient strips (Liofilchem Inc., Waltham, MA) achieved 97% categorical agreement and 93% essential agreement with reference BMD, with no major or very major errors reported (15).

The need to intentionally supplement the cohort with resistant organisms highlights the relative rarity of ATM-AVI resistance among clinical NDM-E isolates. This is consistent with an international collection of 420 NDM-E organisms (78% *K. pneumoniae*) from 27 countries between 2019 and 2022, in which all isolates were susceptible to ATM-AVI (6). Notably, neither this international cohort nor our cohort included isolates from India, the global epicenter of ATM-AVI resistance among NDM-producing *E. coli* (8). For example, among 211 NDM-producing *E. coli* from nine Indian hospitals, over 95% harbored PBP3 insertions, which impact the target of ATM (16). Although these alterations alone may increase ATM-AVI MICs, high-level resistance typically requires concurrent CMY production, which is increasingly reported among *E. coli* isolates from patients with healthcare exposure in India (7, 17). Importantly, PBP3 insertion-mediated resistance appears largely confined to *E. coli*. While NDM production was historically associated with *E. coli*, *K. pneumoniae* now predominates globally among NDM-E. Reassuringly, ATM-AVI appears highly reliable against NDM-producing *K. pneumoniae*, with no published reports of resistance in clinical isolates.

Another key finding was the strong performance of non-reference susceptibility testing methods. Disk diffusion demonstrated excellent categorical agreement with minimal error rates, while both gradient diffusion methods achieved near-perfect categorical agreement. However, essential agreement was lower, and both gradient diffusion methods demonstrated a systematic negative bias, yielding lower MICs than reference BMD. This finding contrasts with observations for several other β-lactams, for which gradient diffusion methods have been reported to yield modestly higher MICs than BMD (18, 19). Notably, however, a similar negative bias has been observed in other evaluations of ATM-AVI gradient diffusion methods (15, 20–22), suggesting that this pattern may reflect characteristics intrinsic to the assay rather than an isolated finding, with the underlying mechanism remaining unclear. Although the observed MIC underestimation did not meaningfully affect categorical interpretation, gradient diffusion MICs should be interpreted with caution when values approach clinical breakpoints.

Several limitations warrant consideration. Despite inclusion of isolates from multiple centers, NDM allele diversity was limited, with a predominance of *bla*_NDM-1_ and *bla*_NDM-7_, which may restrict generalizability to regions with different allele distributions. However, currently available evidence does not suggest an association between specific NDM alleles and ATM-AVI susceptibility or the performance of non-reference testing methods (6, 15). Furthermore, the isolates studied were not derived from patients previously exposed to ATM-AVI; therefore, resistance rates may underestimate those that emerge with broader clinical use.

These limitations notwithstanding, our findings support the promising activity of ATM-AVI against contemporary NDM-E isolates and suggest that both disk and gradient diffusion are reliable alternatives to reference BMD for susceptibility testing, albeit with reduced MIC precision for gradient methods. Continued surveillance is warranted, particularly for the emergence of *E. coli* harboring PBP3 alterations and producing AmpC enzymes.

## Data Availability

The data sets supporting the conclusions of this article are included within the article and its supplemental material.

## AFTER EPUB

*[This article was published on 12 October 2026 with an error in the affiliations. The affiliations were corrected in the current version, posted on 3 November 2026.]*

## SUPPLEMENTAL MATERIAL

Supplemental material jcm.00797-26-s0001.docx Supplemental methods and results.

ASM does not own the copyrights to Supplemental Material that may be linked to, or accessed through, an article. The authors have granted ASM a non-exclusive, world-wide license to publish the Supplemental Material files. Please contact the corresponding author directly for reuse.
