# Supplementary material for: Comparison of antimicrobial susceptibility testing methods for aztreonam-avibactam against NDM-producing Enterobacterales clinical isolates
Source: J Clin Microbiol. 2026 Oct 12;64(11):e00797-26. doi: 10.1128/jcm.00797-26 (PMC13753813; doi:10.1128/jcm.00797-26)
Supplement: Supplementary file 1 [file jcm.00797-26-s0001.docx]

**Comparison of Antimicrobial Susceptibility Testing Methods for Aztreonam-Avibactam Against NDM-Producing Enterobacterales Clinical Isolates:**

**Supplemental Material**

| **Table of Contents** | |
| --- | --- |
| Whole-genome sequencing of a subset of 92 isolates: methods and results | Page 2 |
| **Table S1**: Distribution of sequence types by bacterial species and NDM variant among 92 sequenced isolates | Page 3 |
| **Table S2:** Antimicrobial susceptibility testing results for nine aztreonam-avibactam non-susceptible isolates across testing methods | Page 4 |
| **Table S3**: Bias analysis of gradient diffusion methods compared with broth microdilution for aztreonam-avibactam antimicrobial susceptibility testing | Page 5 |
| **Figure S1:** Geographic distribution of the eight participating hospitals in Chile | Page 6 |
| **Figure S2:** Comparison of aztreonam-avibactam susceptibility results obtained by reference broth microdilution and alternative testing methods | Page 7 |

**Whole-genome sequencing of a subset of 92 isolates**

Whole-genome sequencing data generated through unrelated research studies were available for 92/245 (38%) baseline isolates and all 6 (100%) aztreonam-avibactam (ATM-AVI) resistant isolates using the Illumina platform, enabling identification of specific *bla*_NDM_ alleles. Among the 92 sequenced isolates, 50 and 42 were from Chile and the United States, respectively. Raw sequencing reads underwent quality assessment with FASTQC v0.11.9, with aggregate metrics compiled via MultiQC v1.10.1. Adapter sequences and low-quality bases were removed using FastP v0.23.4. High-quality reads were assembled de novo with SPAdes v3.15.5; assembly integrity was evaluated using QUAST v5.2.0. Genome annotations were generated with Bakta v1.8.2, and sequence types were inferred in silico using MLST v2.23.0. The presence of antimicrobial resistance determinants was identified using AMRFinderPlus v3.11.18.

Among the 92 baseline cohort isolates with sequencing data, NDM alleles included *bla*_NDM-7_ (50/92, 54%) and *bla*_NDM-1_ (42/92, 46%). Co-carriage of carbapenemase genes was identified in three sequenced isolates, including *bla*_NDM-7_ and *bla*_KPC-3_ (1/92, 1%) and *bla*_NDM-7_ and *bla*_KPC-2_ (2/92, 2%). All nine ATM-AVI resistant isolates were *Escherichia coli* and harbored *bla*_NDM-1_, *bla*_NDM-5_, or *bla*_NDM-6_ genes. The ATM-AVI MIC distribution of the nine isolates were as follows:

| ***bla*_NDM_ gene** **(n)** | **Aztreonam (µg/mL)** | | | **Aztreonam-Avibactam (µg/mL)** | | |
| --- | --- | --- | --- | --- | --- | --- |
|  | **MIC_50_** | **MIC_90_** | **Range** | **MIC_50_** | **MIC_90_** | **Range** |
| *bla*_NDM-1_ (42) | >64 | >64 | 16 - >64 | 0.5/4 | 2/4 | 0.06/4 - 4/4 |
| *bla*_NDM-7_ (50) | >64 | >64 | 0.125 - >64 | 0.25/4 | 1/4 | 0.06/4 - 2/4 |

| **Table S1.** Distribution of sequence types by bacterial species and NDM variant among 92 sequenced isolates | | |
| --- | --- | --- |
| ***bla*_NDM_ variant (n)** | **Species (n)** | **Sequence type (n)** |
| *bla*_NDM-7_ (45) | *Klebsiella pneumoniae* (87) | ST45 (30) |
|  |  | ST25 (7) |
|  |  | ST11 (3) |
|  |  | ST528 (3) |
|  |  | New ST (2) |
| *bla*_NDM-1_ (42) |  | ST4843 (29) |
|  |  | ST231 (7) |
|  |  | ST147 (2) |
|  |  | ST11 (1) |
|  |  | ST17 (1) |
|  |  | ST307 (1) |
|  |  | ST3392 (1) |
| *bla*_NDM-7_ (5) | *Enterobacter cloacae* complex (3) | ST45 (1) |
|  |  | ST88 (1) |
|  |  | ST134 (1) |
|  | *Escherichia coli* (2) | ST361 (1) |
|  |  | ST6745 (1) |

| **Table S2:** Antimicrobial susceptibility testing results for nine aztreonam-avibactam non-susceptible isolates across testing methods | | | |
| --- | --- | --- | --- |
| **Broth microdilution (µg/mL)** | **ETEST® (µg/mL)** | **MIC Test Strip™ (µg/mL)** | **Disk diffusion (millimeters)** |
| 8 (I) | 8 (I) | 4 (S) | 25 (S) |
| 8 (I) | 8 (I) | 8 (I) | 22 (I) |
| 16 (R) | 16 (R) | 16 (R) | 24 (I) |
| 8 (I) | 4 (S) | 4 (S) | 23 (I) |
| 8 (I) | 4 (S) | 8 (I) | 24 (I) |
| >64 (R) | 128 (R) | >256 (R) | 12 (R) |
| >64 (R) | >256 (R) | >256 (R) | 19 (R) |
| >64 (R) | >256 (R) | 64 (R) | 12 (R) |
| >64 (R) | >256 (R) | >256 (R) | 20 (R) |
|  |  |  |  |

| **Table S3.** Bias analysis of gradient diffusion methods compared with broth microdilution for aztreonam-avibactam antimicrobial susceptibility testing | | | | |
| --- | --- | --- | --- | --- |
| **Bacterial species** | **Method** | **Positive bias, n (%)** | **Negative bias, n (%)** | **Δbias (%)** |
| All isolates (n=254) | ETEST® | 2 (1) | 82 (32) | -31 |
|  | MIC Test Strip™ | 4 (2) | 63 (25) | -23 |
| *Klebsiella pneumoniae* (n=175) | ETEST® | 0 (0) | 58 (33) | -33 |
|  | MIC Test Strip™ | 1 (1) | 43 (25) | -24 |
| *Enterobacter cloacae* complex (n=51) | ETEST® | 1 (2) | 17 (33) | -31 |
|  | MIC Test Strip™ | 0 (0) | 15 (29) | -29 |
| *Escherichia coli* (n=28) | ETEST® | 2 (7) | 7 (25) | -18 |
|  | MIC Test Strip™ | 2 (7) | 5 (18) | -11 |

**Figure S1.** Geographic distribution of the eight participating hospitals in Chile^1^

^1^Participating institutions are located across three regions (i.e., the Metropolitan, Araucanía, and Los Lagos Regions), providing broad geographic representation of the clinical isolates included in the study. The inset depicts the location of the six hospitals within the Metropolitan Region of Chile.

**Figure S2.** Comparison of aztreonam-avibactam susceptibility results obtained by reference broth microdilution and alternative testing methods^1^

^1^ Reference aztreonam-avibactam (ATM-AVI) broth microdilution (BMD) results were compared with (A) disk diffusion zone diameters, (B) ETEST® MICs, and (C) MIC Test Strip™ MICs.
